# Supplementary material for: qPCR Assay as a Tool for Examining Cotton Resistance to the Virus Complex Causing CLCuD: Yield Loss Inversely Correlates with Betasatellite, Not Virus, DNA Titer
Source: Plants (Basel). 2023 Jul 14;12(14):2645. doi: 10.3390/plants12142645 (PMC10385359; doi:10.3390/plants12142645)
Supplement: Supplementary file 1 [file plants-12-02645-s001.zip › plants-2419252-supplementary.pdf]

plants

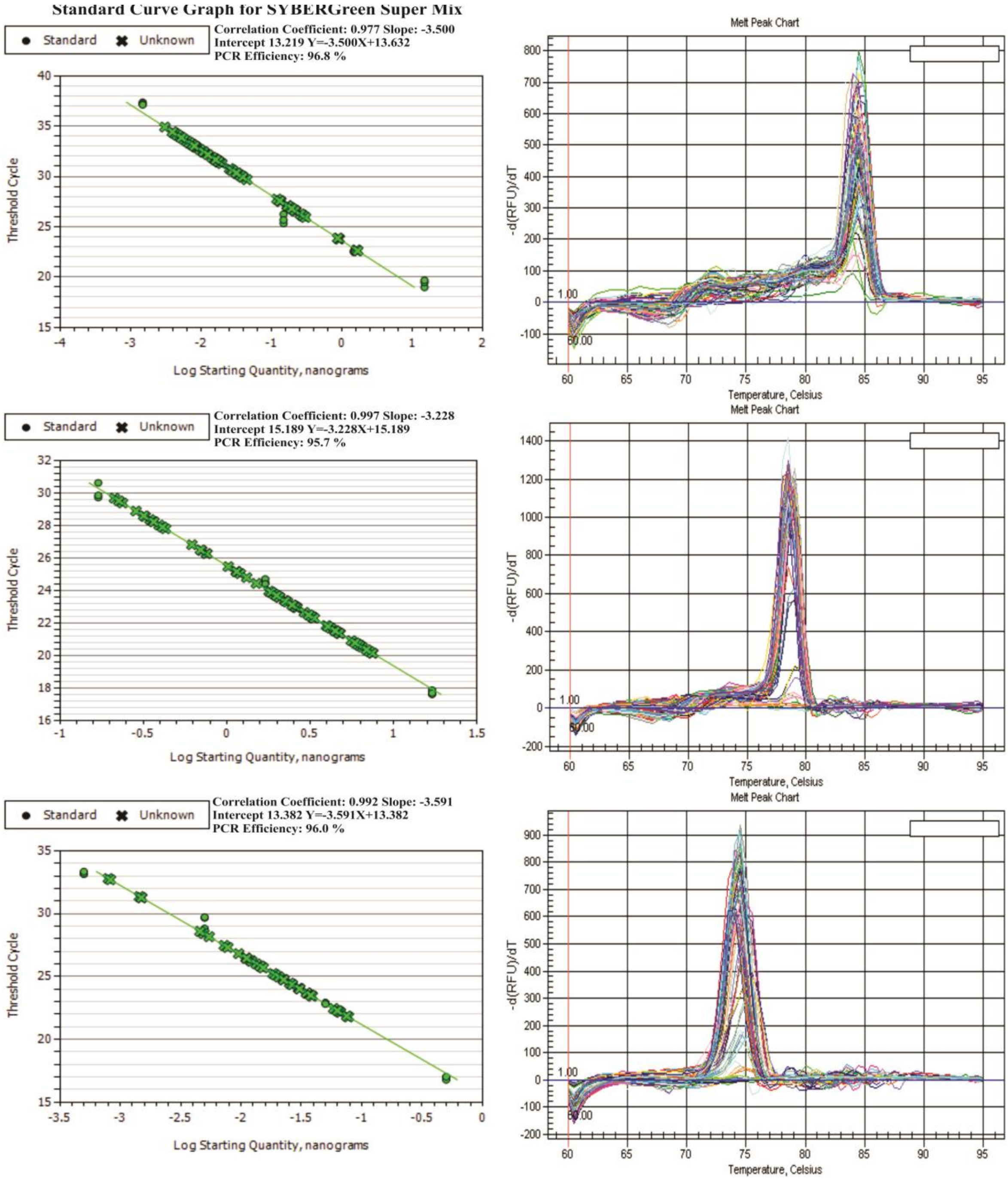

Figure S1. (A) Standard curve for begomovirus with a PCR efficiency of 96.8%, the slope of −3.500 and correlation coefficient 0.977 of begomovirus [left] and melt curve [right]. (B) For betasatellites the standard curve for betasatellite with a PCR efficiency of 95.7%, the slope of −3.228 and correlation coefficient 0.997 [left] and melt curve [right]. (C) Standard curve for alphasatellite with a PCR efficiency of 96.0%, the slope of −3.591 and correlation coefficient 0.992 [left] and melt curve [right]. The melt curve plots the negative rate of change of fluorescence (−d(RFU)/dT) against temperature.
